# Advanced Modification Strategies of Plant-Sourced Dietary Fibers and Their Applications in Functional Foods

**DOI:** 10.3390/foods14152710

**Published:** 2025-08-01

**Authors:** Yansheng Zhao, Ying Shao, Songtao Fan, Juan Bai, Lin Zhu, Ying Zhu, Xiang Xiao

**Affiliations:** School of Food and Biological Engineering, Jiangsu University, Zhenjiang 212013, China; zhaoys@ujs.edu.cn (Y.Z.); 2212318028@stmail.ujs.edu.cn (Y.S.); fansongtao@ujs.edu.cn (S.F.); 1000005134@ujs.edu.cn (J.B.); zhulin19820402@ujs.edu.cn (L.Z.); 1000004369@ujs.edu.cn (Y.Z.)

**Keywords:** plant-sourced dietary fibers, modification, functional foods, gut microbiota

## Abstract

Plant-sourced Dietary Fibers (PDFs) have garnered significant attention due to their multifaceted health benefits, particularly in glycemic control, lipid metabolism regulation, and gut microbiota modulation. This review systematically investigates advanced modification strategies, including physical, chemical, bioengineering, and hybrid approaches, to improve the physicochemical properties and bioactivity of PDFs from legumes, cereals, and other sources. Key modifications such as steam explosion, enzymatic hydrolysis, and carboxymethylation significantly improve solubility, porosity, and functional group exposure, thereby optimizing the health-promoting effects of legume-sourced dietary fiber. The review further elucidates critical structure–function relationships, highlighting PDF’s prebiotic potential, synergistic interactions with polyphenols and proteins, and responsive designs for targeted nutrient delivery. In functional food applications, cereal-sourced dietary fibers serve as a versatile functional ingredient in engineered foods including 3D-printed gels and low-glycemic energy bars, addressing specific metabolic disorders and personalized dietary requirements. By integrating state-of-the-art modification techniques with innovative applications, this review provides comprehensive insights into PDF’s transformative role in advancing functional foods and personalized nutrition solutions.

## 1. Introduction

Dietary fiber, a carbohydrate resistant to digestion enzymes, offers several health benefits, including minimizing blood sugar fluctuations, lowering the risk of diabetes and cardiovascular diseases, maturing the immune system, and supporting regular bowel motions [1]. Plant-sourced Dietary Fibers (PDF) primarily exist as physical complexes of plant tissue particles and intracellular compounds, which are mostly made up of indigestible carbohydrates from plant cell walls [2,3,4]. The physical form of PDF determines the bioavailability of intracellular components, which depends on the porosity and structural complexity of the plant cell wall, as well as the morphology, size, and density of the plant component particles [5]. The health advantages of Plant-sourced Dietary Fibers (PDFs) might not be completely reflected in the experimental results from isolated and purified dietary fibers. According to the available research, intact cell walls can, through an encapsulation mechanism, alter fermentation rates and decrease the bioavailability and extraction efficiency of PDF. Studies on the bioavailability of common pulses through fermentation have shown that the process increases the bioavailability of legumes by disrupting the cell wall’s integrity [6]. Hemery, Y et al. and Hemery, Y.M et al. discovered that lowering the particle size of wheat bran increases the bioavailability of ferulic acid and sinapinic acid [7,8]. Similarly, Bai et al. found that increasing the pH can alter the chemical properties of okra cell walls, causing the cell walls to break, resulting in higher yields and solubility of okra polysaccharides when extracted with alkali [9]. The fact that the molecular weight of polysaccharides from blue honeysuckle with similar structural characteristics strongly influences their anti-gel, anti-glycation, and hypoglycemic in vitro effects also indicates the importance of physical properties on PDF functionality [8].

Based on the solubility of PDF in water, it can be divided into soluble dietary fiber (SDF) and insoluble dietary fiber (IDF). Both SDF and IDF have shown positive physiological effects. Moreover, for metabolic processes like decreasing blood sugar and blood lipids, preventing diabetes and coronary heart disease, and regulating human metabolism, soluble PDF is more advantageous than insoluble PDF [9,10]. In contrast to insoluble PDF, the beneficial health advantages of soluble PDF based on epidemiological studies have pushed the food sector to add soluble PDF into various foods [11,12,13]. Modification is therefore crucial for functional food application since it causes PDFs to undergo physical form changes that improve their bioactive properties. Soluble PDFs can be added to baked goods and other items, helping them act against a variety of illnesses because consumers are more conscious toward their diet and wish for natural remedies [14].

All these studies indicate that the physical structure of PDF and the content of soluble components are more important for its health functions. To investigate the potential contribution of modified PDF to improving the quality and health value of functional food applications, this study examines the impact of modification on the functional properties of plant-derived dietary fiber, as well as the complex relationship between the structure, physicochemical properties of plant-derived dietary fiber and its application in innovative functional foods.

## 2. Sources and Characteristics of PDF

### 2.1. Tissue-Specific Distribution of Dietary Fiber in Plants

**Figure 1:**
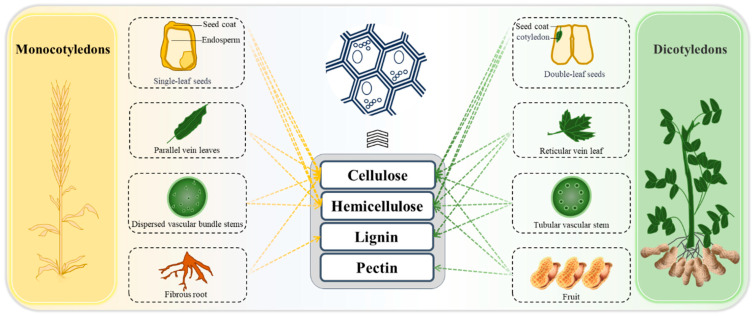
Plant structural origins of Plant-sourced Dietary Fibers.

Plant cell walls serve as the primary source of PDF [15], with its soluble and insoluble components detailed in Table 1. Figure 1 shows that dietary fibers in dicotyledons are primarily distributed within the cell walls of four anatomical structures: seed coats, stems, leaves, and fruits. The seed coats and vegetative tissues (stems and leaves) contain insoluble fibers such as cellulose and lignin that confer structural integrity, while fruit tissues are predominantly composed of soluble fibers including pectin and β-glucans. Notably, pectic polysaccharides and xyloglucans represent the major non-cellulosic polysaccharides in dicotyledonous cell walls. In monocotyledons, dietary fibers mainly localize to the cell walls of seed coats, endosperm, stems, leaves, and roots. Compared with dicotyledons, monocot cell walls exhibit higher arabinoxylan content but reduced abundance of heteroxylans and (1,3;1,4)-β-D-glucans, particularly in economically important poaceae species (cereals and grasses). Notably, mannans, galactomannans, and galactoglucomannans are frequently found in quite high concentrations in the walls of bean endosperm [16].

### 2.2. Physicochemical Properties of PDF

The microstructural modifications in the physical forms of PDF and their spatial arrangement critically influence the structural integrity of the cell wall and the viscoelastic properties of PDF, including solubility variations. For instance, steam explosion technology is widely employed in the modification of PDF sourced from fruits, vegetables, cereals, and nuts [26]. By increasing steam pressure and retention time, this technique reduces the transverse viscosity and mechanical strength of the fiber. During the instantaneous decompression phase, the rapid expansion of pore gases disrupts the crystalline structure of the fiber, thereby enhancing the content of soluble components [27]. Additionally, lignin depolymerization occurs via cleavage of β-O-4 ether linkages and other acid-labile bonds, while simultaneously degrading ester bonds between lignin and phenolic acids. This process liberates insoluble-bound phenolic compounds, ultimately improving their bioavailability [28]. The physical structure of PDF exhibits significant correlations with its physicochemical properties and physiological functionalities. Structural modification of PDF through fiber fragmentation and particle size reduction can effectively enhance its water absorption capacity and swelling properties [29]. Concurrently, increased porosity and increased degree of particle dispersion facilitate the exposure of hydrophobic groups, thereby augmenting oil retention capacity. Furthermore, the formation of a honeycomb-like microstructure significantly improves sodium cholate binding affinity [30], while elevated soluble component content and viscosity effectively retard glucose diffusion rates [31,32].

PDF serves as a crucial modulator of human digestive physiology, exhibiting significant associations with multiple health-promoting effects [33]. Contemporary research has increasingly emphasized the prebiotic potential of PDF in gut microbiota regulation [34,35]. Emerging evidence demonstrates that dysbiosis of gut microbiota is etiologically linked to various metabolic disorders, including inflammatory bowel diseases [36], colorectal inflammation, obesity, and metabolic syndrome [37,38,39]. This has stimulated substantial scientific interest in microbiota-targeted strategies that favor the proliferation of beneficial bacterial taxa [40]. In this context, dietary modulation through PDF supplementation has emerged as an effective nutritional intervention for gut microbial ecosystem remodeling [41,42]. The fermentability of PDF by gut microbiota is highly dependent on its crystallinity, porosity, and bacterial affinity. PDF utilization requires carbohydrate-active enzymes (CAZymes) to cleave specific chemical bonds [43,44]. Linear-structured cellulose exhibits low fermentability due to limited CAZyme accessibility, while branched, low-molecular-weight PDF demonstrates high fermentability [45,46]. For instance, highly fermentable fibers such as inulin [47], both branched and linear forms of Fructooligosaccharides (FOS) [48], and β-glucans [49] have been demonstrated to significantly promote the proliferation of beneficial gut microbiota, particularly *Anaerostipes*, *Bilophila* and *Bifidobacterium.* Concurrently, these fibers exhibit inhibitory effects against potentially pathogenic bacterial populations, including *Bilophila* and *Proteobacteria*. Moreover, PDF with looser, more porous physical structures exhibits greater accessibility to both enzymes and gut microbiota. A representative example is wheat bran arabinoxylan (AX), which preferentially enriches butyrate-producing *Faecalibacterium* [50].

## 3. Innovative Modification Techniques for PDF

For the high-value sustainable utilization of PDF, pretreatment is essential to disrupt their relatively dense physical structural barriers, thereby facilitating efficient extraction, conversion, and utilization of PDF.

### 3.1. Physical Modification

The glycosidic bonds of PDF can be modified through physical methods such as high temperature, high pressure [51], instant pressure release, ultrasound [52], explosion, high-speed impact, and shear force [53], which melt or break these bonds. Cavitation jets [54], a highly efficient fragmentation method utilizing high temperature, high pressure, and high shear force to alter intermolecular hydrogen bonds and crystalline structures of PDF, effectively reduces particle size, increases amorphous structures, and enhances soluble components, thereby improving its physicochemical and functional properties. For instance, Wu et al. applied cavitation jets to okara dietary fiber, significantly increasing SDF content and yielding higher shear viscosity [55].

Steam burst pretreatment is another emerging, efficient, and green modification technology for food ingredients. It relies on the rapid expansion of steam within material voids, inducing mechanical fracture and hydrogen bond disruption. This process cleaves glycosidic bonds and hydrogen bonds between lignin, cellulose, and hemicellulose, explosively rupturing cell walls into porous, low-molecular-weight substances [56]. This method offers advantages such as broad applicability, environmental safety, and scalability for industrial production. Steam pretreatment combined with hot water extraction effectively isolates hemicellulose from bamboo shavings, significantly enhancing phagocytic activity and nitric oxide (NO) production in murine macrophage RAW264.7 cells, thereby improving immunomodulatory potency [57].

High hydrostatic pressure (HHP) and superfine grinding (SFGT) are widely adopted as efficient modification techniques for developing functional fiber ingredients, owing to their advantages in performance, cost-effectiveness, and sustainability. These methods have been extensively applied to modify dietary fibers derived from various fruits and vegetables. For instance, Ling et al. investigated the effects of HHP and SFGT on the physicochemical and functional properties of pear pomace [58]. SFGT’s intense mechanical shearing disrupts chemical bonds in pear pomace, increasing soluble component content, while HHP alters the internal structure of fiber cells and cell membranes, enhancing functional properties such as water-holding and oil-binding capacities.

Compared with conventional spray-drying for food encapsulation, nanofibrous membranes of soluble cellulose produced by electrospinning technology exhibit superior stabilization effects on bioactive compounds, demonstrating great potential as novel delivery carriers for food ingredients [59,60]. The electrospinning nanofibrous membranes fabricated from PDF exhibit remarkable enhancement in both encapsulation efficiency and bioavailability of target bioactive compounds [61]. These advanced fibrous matrices, characterized by their outstanding biocompatibility and tunable biodegradability, represent a novel class of delivery systems with significant potential for the nano/microencapsulation of functional food ingredients [62,63]. Guar gum, a neutral polysaccharide extracted from leguminous plants, consists of supramolecular aggregates of galactomannan with multi-chain structures. After electrospinning treatment, the molecular aggregates of guar gum were effectively eliminated, and the local interchain coupling was disrupted, resulting in reduced fiber diameter and enhanced fiber uniformity. The resultant nano-guar gum films exhibited superior toughness and improved biodegradability [64].

### 3.2. Sustainable Chemical Modification

Chemical modification effectively depolymerizes PDF macromolecules, disrupts dense networks, and cleaves glycosidic bonds to generate reducing terminals. The chemical modification of PDF is engineered via acid/alkali-mediated structural modulation, or covalent functionalization (e.g., esterification) to tailor physicochemical properties [65,66].

Common acids used for chemical modification of PDF include hydrochloric acid, citric acid, malic acid, and lactic acid, with NaOH being the most frequently used alkali. When wheat bran undergoes modification using NaOH, epichlorohydrin, and dichloromethane, the resulting fibers develop porous, irregular structures with modified crystalline patterns and newly introduced active groups that collectively enhance adsorption capacity, creating an effective and environmentally friendly agent for wastewater dye removal [67]. FTIR analysis of hydrochloric acid-modified wheat bran showed significantly reduced absorption peak intensity at C-O stretching vibrations, indicating degradation of cellulose and lignin components. The increased soluble fraction content led to a more porous structure, exposing additional active groups that improved antioxidant capacity [68]. However, while controlled acid/alkali treatments effectively modify dietary fiber through microstructure disruption and polymerization degree reduction, excessive processing conditions must be carefully avoided to prevent undesirable macromolecular degradation and preserve functional properties [69].

Compared to the FTIR spectrum of native cellulose, carboxymethylated cellulose (CMF) exhibits characteristic peaks corresponding to -CH_3_ symmetric vibration and C-O-C stretching vibration, confirming the successful incorporation of carboxylate groups [70]. Similarly, carboxymethyl groups were detected in carboxymethylated goji berry pomace, consistent with the C=O stretching vibration in FTIR spectra [71]. Carboxymethylation of banana fibers resulted in reduced crystallinity, a more porous and looser fibrous structure, and an increased specific surface area, which significantly enhanced their heavy metal adsorption capacity [72]. The modified PDF developed a porous surface structure, exhibiting a high proportion of SDF and remarkable water-swelling ability. These modifications not only increased the viscosity of aqueous solutions but also endowed the PDF with superior cholesterol adsorption and bile salt binding capacities [73].

Acetylation primarily occurs on the side chains of polysaccharides and serves as an effective method to enhance the hydrophobicity of PDFs. By substituting hydrophilic hydroxyl groups with acetyl groups, acetylation alters the molecular chain orientation and polysaccharide conformation of PDFs while exposing additional polar groups to improve water solubility [74]. Recent studies have demonstrated that acetylated millet bran exhibits a significant increase in SDF content, forming a porous microstructure that enhances its sodium cholate and nitrite ion adsorption capacities [31]. Post-acetylation, the fiber’s surface morphology undergoes noticeable changes, accompanied by reduced crystallinity [75]. Furthermore, FTIR spectroscopy reveals C-H asymmetric stretching and carbonyl bending vibrations, confirming the successful introduction of acetyl groups into millet bran [31]. However, excessive acetylation of fibers may enable functional fiber-fortified foods to bind minerals such as calcium, iron, and zinc through carboxyl groups, thereby reducing their bioavailability and impairing fermentation by gut microbiota including *Bifidobacterium* and *Lactobacillus*. Prolonged consumption may consequently affect nutritional balance. Therefore, stringent process control is required for functional foods containing acetylated fibers, particularly for safety-sensitive products like infant foods.

The targeted introduction of functional groups to achieve specific physiological benefits has gained significant attention in medical therapeutic applications. Sulfate esterification alters sugar ring conformation, where mutual repulsion between similarly charged groups induces an extended chain configuration from originally coiled structures. This structural transformation is clearly observed in SEM images of both sulfate esterification seedless pear pomace dietary fiber and corn bran polysaccharides, which exhibit more ordered architectures with uniformly smooth surfaces [76]. FTIR spectroscopy confirms successful sulfation through S=O stretching vibration and C-O-S stretching vibration, demonstrating covalent incorporation of sulfate groups into PDF [77]. Importantly, gene expression analysis of tumor-suppression-related markers revealed that sulfated rice bran polysaccharides exhibit significant antitumor activity against HEP-G2 and B16 cell lines, demonstrating the considerable potential of sulfated polysaccharides in cancer intervention strategies [78]. Notably, sulfated fibers readily decompose at high temperatures (120 °C), releasing irritant SO_2_, while excessive intake may alter intestinal pH and consequently induce gut microbiota dysbiosis. Therefore, precise dosage control and stringent processing standards must be implemented prior to incorporating sulfated fibers into functional foods.

### 3.3. Bioengineering Modification

The bioengineering of PDF refers to the process of directed modification, synthesis, or optimization of dietary fibers using genetic engineering, enzymatic processing, fermentation [79], and related biotechnological approaches. This process aims to enhance their functional properties, bioavailability, and health-promoting effects.

Enzymatic modification primarily involves the use of cellulase and xylanase to degrade cellulose and hemicellulose, thereby increasing the SDF content and enhancing the functional properties of PDF. Liu et al. [80] investigated the cellulase-mediated modification of rice bran dietary fiber and found that the treated fiber exhibited increased porosity and reduced crystallinity due to hydrogen bond disruption [81]. Cellulase treatment resulted in a honeycomb-like network structure with abundant cavities, which not only provided additional space for water molecule storage via hydrogen bonding and dipole interactions but also facilitated more efficient cholesterol binding [82]. FTIR spectroscopy revealed broad and flattened absorption peaks, indicating that hydroxyl groups generated by cellulase hydrolysis promoted the cleavage of glycosidic bonds in the dietary fiber structure, thereby accelerating the degradation of small-molecular-weight sugars [81]. In contrast, xylanase can hydrolyze the insoluble arabinoxylan backbone in wheat bran, thereby increasing its SDF content. Furthermore, enzymatic modification reduced particle size, releasing bound phenolic compounds and other bioactive substances, ultimately improving the nutritional and functional quality of wheat bran [83].

Fermentation serves as a cost-effective and eco-friendly strategy for modifying PDF, effectively converting it into SDF while enhancing its functional properties [84,85]. Microbial selection critically influences PDF’s structural and bioactivity modifications [86,87]. For example, *Bacillus subtilis* fermentation of okara exhibits high cellulase activity, disrupting crystalline structures and releasing oligosaccharides via lignocellulose degradation [88]. Co-fermentation with lactic acid bacteria and *Kluyveromyces marxianus* significantly improves the hypoglycemic and hypolipidemic potential of okara IDF [89]. Meanwhile, *Trichoderma viride* enzymatically hydrolyzes defatted rice bran IDF, yielding SDF with enhanced solubility, surface-active groups, and improved water/oil-holding capacities [90]. FTIR spectroscopy indicates depolymerization of polysaccharide chains (peak shifts to lower wavenumbers, increased intensity) [91], while exposed hydroxyl groups and porous structures enhance free radical scavenging [92]. Additionally, strengthened hydrophobic and van der Waals interactions elevate cholesterol adsorption and pancreatic lipase inhibition [89].

While cellulase-producing strains such as *Bacillus subtilis* exhibit potential for dietary fiber modification, their native cellulase systems are inherently incomplete. Notably, the absence of exoglucanase-encoding genes significantly impairs their capacity to efficiently disrupt the crystalline structure of PDF. To overcome this limitation, genetic engineering approaches have been developed to enable heterologous overexpression of cellulase genes in *B. subtilis*, thereby substantially enhancing its cellulose degradation efficiency [93]. Moreover, the introduction of cellobiose dehydrogenase (CDH) genes into *Aspergillus niger* has demonstrated remarkable effects. CDH catalyzes the hydrolysis of cellobiose, lactose, and cello-oligosaccharides into carboxylic acids, resulting in a 28.57% increase in lignocellulolytic activity and significantly improved bioconversion rates [94]. These engineered microbial strategies represent promising avenues for targeted modification of PDF to meet the growing demands of functional food.

### 3.4. Hybrid Modification

Hybrid modification strategically integrates multiple techniques to address the inherent constraints of single-method approaches, thereby co-optimizing PDF production yield and functional quality. Recent studies demonstrate that high-temperature cooking effectively cleaves glycosidic bonds, facilitating the conversion of IDF to SDF. Subsequent ultrasonic-assisted enzymatic treatment further enhances SDF yield, surpassing the efficiency of individual modification approaches [95]. Similarly, while high-temperature cooking and cellulase treatment alone produce spherical microstructures in mung bean hull dietary fiber, their combined application generates porous lamellar structures. These structural modifications expose additional hydrophilic groups, significantly improving water solubility, water-holding capacity, and oil-holding capacity. The enhanced thermostability and physicochemical properties correlate with beneficial physiological effects, including reduced serum cholesterol levels, improved glucose adsorption capacity, and enhanced intestinal fermentability [96].

Enzyme–microbial synergistic fermentation has garnered considerable research attention due to its environmental friendliness and minimal byproduct formation. Guo et al. demonstrated that combined enzymatic (cellulase and xylanase) and microbial (*Lactobacillus plantarum* and *Bifidobacterium*) modification of sea buckthorn increased amorphous structures and promoted the release of active groups [97]. This process significantly enhanced the binding capacity of negatively charged phenolic acid groups with NO^2-^, thereby improving nitrite-scavenging activity. Comparable structural modifications were observed in *Aspergillus niger*-cellulase co-treated *Mesona chinensis* Benth residue dietary fibers, where cross-linked molecular rearrangement generated more pronounced amorphous structures than enzymatic treatment alone [98]. The resulting honeycomb-like microstructure enhanced adsorption properties, substantially increasing the economic value of this agricultural byproduct.

### 3.5. Comparative Analysis of Modification Methods

Physical modification offers advantages such as simplicity and low cost, but excessive mechanical shear force can disrupt the crystalline structure of fibers, leading to the breakage of dietary fiber molecular chains and reduced hydration capacity [22]. Compared to physical modification, chemical modification can effectively control the solubility of dietary fibers; however, residual chemical reagents may pose food safety risks, and the required purification steps increase production costs [99]. In contrast to physical and chemical modifications, biological modification operates under mild conditions, offering eco-friendliness and specific catalytic advantages. Nevertheless, the substrate sensitivity of enzymes limits the modification efficiency of dietary fibers, while the high cost of enzymes, stringent reaction conditions, and technical complexity present additional challenges.

Combined modification strategies can compensate for the limitations of individual modification methods and meet diverse modification requirements [100]. While combined modification demonstrates significant advantages in functional optimization of dietary fibers, existing combined modification processes remain technically complex [101]. Moreover, potential interference between different modification methods may compromise the overall modification efficacy. Therefore, developing novel systematic modification approaches by evaluating the interplay between modification effects, safety considerations, cost factors, and application demands is crucial for advancing combined modification technologies.

## 4. Mechanisms of Functional Improvement in Modified PDF

**Figure 2:**
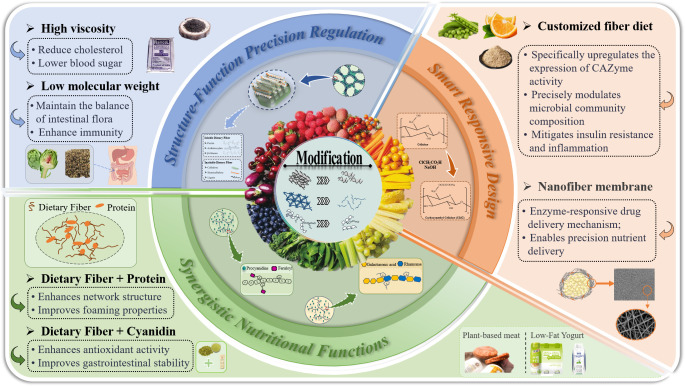
Mechanisms of functional enhancement in Plant-sourced Dietary Fibers for functional food applications.

The above discussion confirms that the physical structure of dietary fibers critically determines their functional activity. Moreover, different modification methods lead to distinct structural alterations in PDF [83]. Consequently, targeted structural modulation of PDF can enhance specific functionalities to meet diverse nutritional requirements, thereby driving revolutionary advancements in functional foods (Table 2). Figure 2 summarizes the functional enhancement mechanisms of modified plant-based dietary fibers in food applications, primarily including precise structure–function regulation, synergistic nutrient interactions, and smart responsive designs for precision nutrition.

### 4.1. Structure–Function Precision Regulation

#### 4.1.1. Modified Plant-Sourced Dietary Fiber Viscosity

The viscosity of dietary fiber is a critical mechanism influencing human health. High-viscosity fibers like psyllium and guar gum primarily offer cholesterol-lowering and glycemic control benefits, while low-viscosity soluble fibers (e.g., inulin and fructooligosaccharides) are more readily fermented by gut microbiota to produce SCFAs, thereby enhancing systemic immunity and intestinal barrier integrity. For instance, enzymatic hydrolysis by cellulase reduced the molecular weight of sunflower seed pectin from 800 kDa to 12.5 kDa, and artichoke pectin from 500 kDa to 80 kDa. The enzymatically modified pectins demonstrated preferential enrichment of gut *Bifidobacterium* and *Lactobacillusv* [111]. Fiber viscosity also determines its food applications. Lower-molecular-weight PDF from mung bean hull (45 kDa) demonstrates stronger hydroxyl radical-scavenging activity [112]. In orange juice, PDF disrupts the expanded network structure, increasing water solubility. In addition, the breakdown of PDF internal architecture reduces viscosity, improving fluidity and significantly diminishing gel-like properties. The decreased shear modulus yields a clearer juice texture, better meeting the sensory preferences of children and elderly consumers [113].

#### 4.1.2. Modified Plant-Sourced Dietary Fibers with Hierarchical Porosity

The highly porous network microstructure endows PDF with superior adsorption capacity and enhanced probiotic colonization rates. When rice bran dietary fiber undergoes high-pressure homogenization modification, the disruption of hemicellulose and increased amorphous regions result in reduced crystallinity, forming a microporous structure with exposed hydrophilic groups. These functional groups exhibit ion-exchange capacity for Ca^2+^, Zn^2+^, and Cu^2+^, maintaining stable pH and ionic balance to improve both the palatability and nutrient bioavailability of PDF-fortified foods [81]. Juodeikiene, G. et al. demonstrated that ultrasonic treatment of soya press cake (okara) significantly increased porosity, elevating the SDF content from 2.896 to 2.963 g/100 g, while simultaneously providing an optimal nutrient matrix for lactic acid bacteria to enhance L-lactic acid production [114].

### 4.2. Synergistic Nutritional Functions

#### 4.2.1. Polyphenol-Bound Plant-Sourced Dietary Fiber Complexes

Plant polyphenols are secondary metabolites characterized by a phenolic skeleton and abundant hydroxyl groups, exhibiting diverse biological activities such as cardiovascular disease prevention, anti-inflammatory effects, and anticancer properties [115,116]. The presence of numerous weakly acidic hydroxyl groups in polyphenols enhances the stability of fiber–polyphenol complexes in acidic environments. Compared to fiber alone, these complexes more readily bind hydrogen atoms from unsaturated bonds, thereby improving oil adsorption capacity, antioxidant activity, and the ability to scavenge reactive carbonyl species [117,118,119]. Moreover, multiple studies have demonstrated that PDFs can protect polyphenols during delivery to colonic target sites, where they are metabolized by gut microbiota to exert health benefits, thereby enhancing the bioaccessibility and bioavailability of polyphenols [120]. For instance, Lu et al. investigated the impact of dietary fiber–phenolic compound complexes on gut microbiota using an in vitro colonic fermentation model [121]. The results revealed that the unabsorbed complexes in the upper digestive tract were primarily fermented into dominant bacterial phyla, including *Firmicutes* and *Bacteroidetes*, and exhibited superior modulation of gut microbial diversity compared to polyphenols alone. In contrast to natural cellulose, nanocellulose provides more binding sites, forming complexes with stronger antioxidant activity and sustained release properties. Li et al. compared nanocellulose–quercetin complexes with quercetin, demonstrating that nanocellulose effectively preserved the stability of quercetin throughout the gastrointestinal tract [121].

#### 4.2.2. Protein-Bound Modified Plant-Sourced Dietary Fiber Complexes

Gelation represents an effective strategy for modulating textural characteristics in food systems, particularly in dairy and processed meat products, to address contemporary nutritional and health demands [122]. The formation of biopolymer complexes between PDF and proteins typically yields superior mechanical properties and enhanced structural stability compared to individual components [123]. Notably, covalently conjugated polysaccharide–protein complexes exhibit a distinctive lamellar and microporous architecture, wherein polysaccharide incorporation not only increases protein matrix flexibility but also significantly improves both foaming characteristics and gel network strength [124]. A representative example involves the Maillard reaction products (MRPs) generated between soy protein isolate (SPI) amino groups and α-lactose monohydrate carbonyl moieties. The emulsion stability, emulsifying activity, foaming capacity, foaming stability, and gel strength of these conjugates increased by 259%, 55.71%, 82.32%, 58.53%, and 3266%, respectively. This enhancement was attributed to the introduction of additional hydrophilic groups, as the covalent attachment of saccharide residues to SPI enhances protein solubility by exposing more free amino groups, thereby optimizing surface-active properties [125]. Parallel observations were made in oat β-glucan–SPI conjugates, where Fourier-transform infrared spectroscopy revealed decreased N-H bending vibrations (amide II band), confirming successful cross-linking. Importantly, the degree of cross-linking exhibited positive correlation with β-glucan content, suggesting progressive SPI amino group engagement with polysaccharide carbonyls. This molecular interaction elongates the SPI polypeptide chain, ultimately enhancing both aqueous solubility and gel-forming capacity [126]. These findings underscore the potential of strategically modified Plant-sourced Dietary Fiber–protein complexes as functional ingredients in precision nutrition applications, particularly for designing foods with tailored textural and nutritional profiles.

### 4.3. Smart Responsive Design

Smart responsive design of dietary fibers refers to the targeted modification of fibers based on their utilization patterns and sites of bioactivity manifestation. This approach aims to achieve targeted functionality in the gastrointestinal tract or food matrices, optimizing both technological performance and health benefits.

#### 4.3.1. Smart pH-Sensitive Modification of Plant-Sourced Dietary Fibers

Smart responsive design of PDF refers to the molecular engineering and functional modification of dietary fibers to enable controlled responsive behaviors to environmental stimuli (e.g., pH, enzymes, temperature, ionic strength, or gut microbiota) [127,128]. This allows for precise functional regulation in food processing, nutrient delivery, or health interventions [129,130,131]. The carboxyl groups in carboxymethyl cellulose (CMC) exhibit distinct states under varying pH conditions. Under alkaline conditions (pH = 9), the carboxyl groups deprotonate, adopting a negatively charged state, with swelling ratios ranging from 308.03% ± 2.36 to 596.24% ± 1.22. Conversely, under acidic conditions (pH = 1.2), protonation occurs, resulting in reduced swelling capacity, with swelling ratios ranging from 152.25% ± 1.49 to 210.22% ± 1.42 [132,133,134]. Furthermore, the enzymatic degradation of cellulose by cellulase enables the development of diverse enzyme-responsive drug delivery systems [135]. For instance, CMC can be selectively hydrolyzed by cellulase, thereby triggering the controlled release of encapsulated therapeutics [136].

#### 4.3.2. Combinatorial Customization of Modified Dietary Fiber for Personalized Gut-Targeted Delivery

Prolonged excessive intake of a single type of dietary fiber may induce intestinal microbiota dysbiosis, which can be mitigated through strategic fiber blending approaches. A closed-loop regulatory strategy for personalized nutritional intervention was developed by tailoring fiber blends based on individual microbiota signatures [137]. Fecal microbiota from obese donors was transplanted into germ-free mice, followed by alternating supplementation with 10% pea fiber, orange fiber, or barley bran under a diet high in saturated fats and low in fruits and vegetables (HiSF-LoFV). Key findings revealed that pea and orange fibers significantly enriched *Bacteroide* and activated metabolic pathways for arabinose and galacturonic acid, while barley bran preferentially upregulated β-glucanase gene expression and enriched xylan-degrading bacteria. The multi-fiber blend synergistically enhanced carbohydrate-active enzyme (CAZyme) activity and exhibited lower interindividual variability in microbial responses compared to single fibers. Highly consistent functional microbiota responses were further validated in human trials. This microbiota-directed approach enabled the design of targeted fiber combinations to modulate microbial functions, with the optimized blend demonstrating efficacy in alleviating insulin resistance and low-grade inflammation-associated dysbiosis [138].

Moreover, omics-driven strategies can be employed to achieve precision fermentation for fiber customization, representing a future direction for modifying PDF. Initially, genome-wide association analysis (GWAS) of plants with high-functionality fiber content can identify key biosynthetic genes. Studies have demonstrated that integrating quantitative genetics with transcriptomic data enables selection of optimal candidate genes for breeding plants with superior dietary fiber profiles [139]. Subsequently, by analyzing cellulosease genes correlated with short-chain fatty acid (SCFA) production and predicting the metabolic networks of gut microbiota toward modified fibers through metagenomics, detailed glycomic characterization of PDF can be integrated with gut microbial and metabolomic data. Maldonado-Gomez Maria, X. et al. demonstrated through their research on the regulatory effects of structurally distinct fibers on human gut microbiota that oat, barley, and rye fibers composed of specific glycans can selectively drive butyrate production, with higher fiber content leading to elevated butyrate levels. This structure-dependent modulation of gut microbiota by dietary fibers enables the artificial customization of optimally fermentable PDF structures [140], providing a cutting-edge approach for developing functional food products with targeted health benefits.

## 5. Main Approaches for Functional Food Applications of PDF

### 5.1. Engineered Fiber Formulation Design

#### 5.1.1. Low-Calorie Functional Foods

Rapid urbanization and economic development have led to reduced physical activity and lifestyle changes, with unhealthy diets being a major contributor to the rising prevalence of non-communicable diseases (NCDs) such as diabetes, cardiovascular diseases, and cancer. To mitigate NCD risks, reducing sugar and caloric intake is strongly recommended. The incorporation of low-viscosity pectin into yogurt beverages enhances textural viscosity while reducing subjective hunger perception, thereby exerting an appetite-suppressing effect. Studies have demonstrated that consumption of a low-calorie beverage supplemented with 8 g of low-viscosity pectin fiber significantly reduces energy intake during the subsequent meal. Moreover, compared to beverages consumed 90 min before the test meal, those ingested 15 min prior elicited greater satiety and lower subsequent energy intake [141,142].

β-Glucan exhibits exceptional thickening, emulsifying, and gelling properties, endowing it with potential as a key ingredient in the food industry. The incorporation of β-glucan enhances beverage viscosity and color stability while modifying the structure, texture, and sensory acceptability of baked goods [143]. Relevant studies demonstrate that yogurt supplemented with 0.4% (*w*/*w*) barley-derived β-glucan and yogurt containing 0.2% (*w*/*w*) citrus peel pectin exhibit superior proteolytic activity, which significantly improves bioaccessibility during in vitro-simulated gastric digestion. This is evidenced by increased soluble protein content from 25% to 34% and 35%, respectively. Furthermore, compared to control yogurt without dietary fiber supplementation, the β-glucan-fortified yogurt showed enhanced release of free amino acids (FAAs) during simulated gastric digestion, increasing from 3 to 8 mg/protein, consequently improving gastrointestinal absorption and nutrient bioavailability [144]. Additionally, incorporating polydextrose as a fat replacer and resistant starch to optimize the texture and sensory properties of low-fat biscuits enables the production of high-fiber, low-calorie multigrain biscuits with a soft yet crisp texture [145]. This approach aligns with growing consumer demand for nutrient-dense functional foods [146].

#### 5.1.2. Microbiota-Targeting Functional Foods

Furthermore, dietary intake of PDF can exert hypoglycemic, hypolipidemic, and hypotensive effects through stimulated colonic fermentation [147,148,149]. Supplementation with anthocyanin-prebiotic (rice bran soluble fiber and inulin) formulations in type 2 diabetes mellitus (T2DM) patients effectively delays glucose absorption and modulates serum lipid profiles [150]. Relevant studies indicate that propionate and butyrate, produced by gut microbial fermentation of SDF, primarily activate intestinal gluconeogenesis (IGN) gene expression via the gut–brain neural circuit, thereby maintaining glucose and energy homeostasis [151].

### 5.2. Innovative Product Applications

#### 5.2.1. 3D-Printed Personalized Nutrition Foods

The utilization of 3D printing to process PDF-based foods enables the production of customized engineered foods tailored to individual characteristics and nutritional requirements [152]. Since the addition of IDF at levels >5% may impart undesirable textural roughness to food products, while soluble fibers exceeding 3% could lead to excessive viscosity and sliminess, it is essential to optimize their incorporation levels in food formulations. Only through such precise dosage control can the optimal physiological functionality of dietary fibers be effectively realized. For instance, cod protein composite gels (CPCGs) formulated with inulin, soybean SDF, flaxseed oil, and homogenized steamed cod demonstrated that, when maintaining identical flaxseed oil content with a 5:5 ratio of inulin to SDF, the hardness, chewiness, and viscosity of CPCG were significantly reduced while its swallowability was improved. Furthermore, SDF physically embedded within the gel matrix, resulting in a more compact CPCG network structure that enhanced the printing precision and stability of the 3D-printed composite hydrogel [153].

Compared to conventional market-available biscuits, 3D-printed biscuits exhibit superior acceptability due to their esthetic versatility, enhanced crispness, and optimized flavor profiles. Okara fiber modified through the synergistic combination of 500 W ultrasonic treatment and high-speed homogenization at 15,000 rpm demonstrated optimal swelling capacity, water-holding capacity (WHC), and oil-holding capacity (OHC). When incorporated into dough at 6% addition level, the modified okara fiber produced cookie dough with reduced hardness, enhanced elasticity, and maximum adhesion force, ultimately yielding cookies with optimal 3D-printing performance. Furthermore, the abundant hydrophilic groups in the dietary fiber contributed to improved crispness of the final product. The PDF-modified 3D-printed cookies also exhibited diversified flavors and shapes, which not only cater to modern consumers’ sensory preferences but also show potential for obesity mitigation through controlled nutrient delivery [154].

#### 5.2.2. Low-Glycemic-Index (GI) Energy Bar

Energy bars represent a convenient and nutritionally complete food category with demonstrated physiological regulatory effects. Substituting refined carbohydrates and fats in traditional energy bars with PDF and protein modulates meal-associated energy intake while improving short-term glucose and insulin homeostasis [155,156].

The incorporation of kale at 30% (*w*/*w*) into multigrain bars significantly improved their physicochemical properties: hardness decreased from 298.2 N to 94.6 N, chewiness was reduced by approximately six-fold, antioxidant activity increased from 23.6 mM trolox equivalents/g dry matter (d.m.) to 29.3 mM trolox equivalents/g d.m., and total polyphenol content rose from 334 mg gallic acid equivalents/100 g d.m. to 422 mg gallic acid equivalents/100 g d.m. The resulting product not only provides rapid satiety but also delivers naturally beneficial bioactive compounds [157]. For athletic populations, consumption of low-glycemic-index (GI) nutritional energy bars promotes fat oxidation while reducing carbohydrate oxidation, leading to measurable improvements in running and jumping agility. Controlled studies simulating soccer match conditions demonstrate that athletes consuming lentil-based, low-GI nutrition bars during halftime exhibit reduced carbohydrate oxidation rates, thereby preserving glycogen stores. This metabolic adaptation correlates with enhanced second-half performance metrics, including sprint speed, heading accuracy, and total distance covered [158].

### 5.3. Sensory Properties and Consumer Acceptance

The poor solubility and weak water-holding capacity of PDF often result in inferior quality characteristics in processed foods, including inadequate mechanical properties, loose structure, and coarse texture, thereby limiting its broader application in food processing [159]. To address these challenges, texture modification of PDF is essential to alter its physicochemical properties and ultimately enhance the palatability of high-fiber food products [160]. Notably, corncob fermented with Ganoderma lucidum exhibits a significant increase in SDF content compared to unfermented corncob. This modification leads to dough with higher free phenolic content and total antioxidant capacity, improving its extensibility, rheological properties, and leavening ability. Consequently, the resulting gluten-free breadsticks demonstrate increased breaking force and enhanced baking flavor profiles [161,162].

Cakes and cream fillings are significant contributors to food flavor and caloric content, primarily due to their high fat composition. While fats impart unique textural and functional properties to food products, low-calorie cream fillings formulated with fat replacers such as microcrystalline cellulose and maltodextrin can achieve comparable rheological and mechanical characteristics [163,164,165]. As an alternative fat substitute, PDF not only replicates these desirable properties but also offers additional health benefits, including improved digestibility and reduced risks of chronic diseases such as cancer and cardiovascular disorders. This positions PDF as a sustainable and cost-effective supplement that aligns with the food industry’s growing demand for low-fat and low-sugar products [166]. Notably, lemon fiber—enriched with bioactive compounds like flavonoids, vitamin C, and phenolic acids—exhibits exceptional water- and oil-holding capacities, along with significant colon fermentation potential [167]. Incorporating lemon fiber into cream fillings increases viscosity, enhances creaminess, and mimics the rheological and textural properties of traditional shortening. Remarkably, this substitution reduces fat content from 30% to 10%, meeting consumer preferences for low-fat, functional, and clean-label products [168].

Furthermore, the content and pre-/post-modification safety of PDF represent critical metrics for functional food applications. PDF modifications must comply with the GRAS certification requirements (FDA 21 CFR 170.30) for novel processing methods [169,170]. When using processing aids such as sodium hydroxide, enzymatic preparations (e.g., papain, lipase), or ethanol during modification, their types, quality specifications, and final residual levels must adhere to GB 2760 ‘Standards for Use of Food Additives’ [171].

## 6. Conclusions

**Figure 3:**
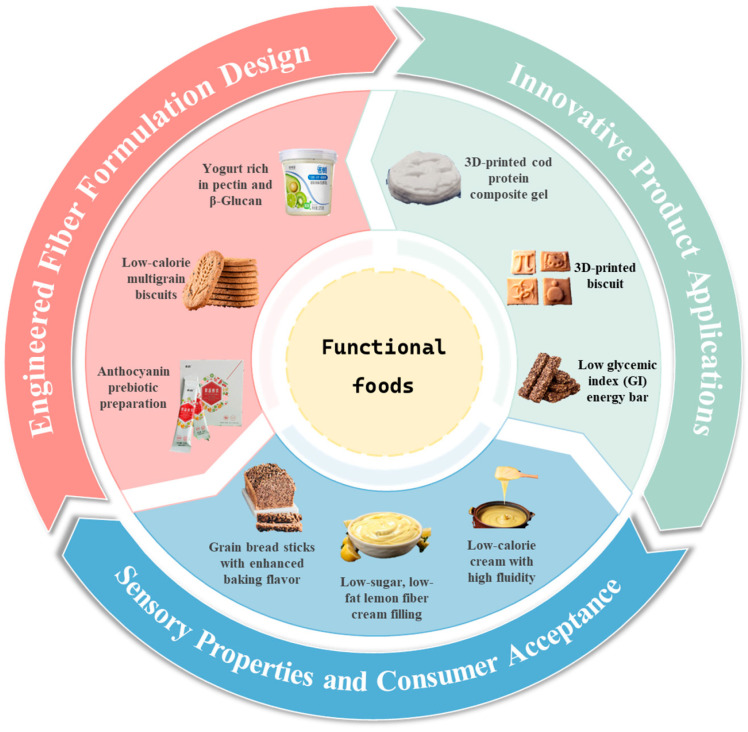
Applications of Plant-sourced Dietary Fibers in functional foods.

This review focuses on innovative modification strategies for PDF and how these approaches uniquely enhance both nutritional and sensory properties in functional foods (Figure 3), representing a dual improvement that addresses key limitations of conventional fiber fortification. The transition from simple PDF addition to intelligent, structure-driven PDF design demonstrates potential for precise nutritional regulation, laying the foundation for precision nutrition applications.

Compared with existing methods, these innovative modification strategies enable texture customization and superior bioactive compound encapsulation without flavor compromise, representing a crucial solution to major industry challenges. Furthermore, PDF incorporation into functional foods through structure–function relationships can provide targeted health benefits. These findings offer researchers and food manufacturers novel pathways to develop next-generation functional foods, which are particularly valuable for addressing nutrition-related non-communicable diseases in developing regions.

## Figures and Tables

**Table 1:** Dietary fiber content from different plant sources.

Plant Sources	Total Dietary Fiber (%)	Soluble Dietary Fiber (%)	Insoluble Dietary Fiber (%)	Refs.
Wheat bran	59.10 ± 0.2	15.50 ± 0.30	43.50 ± 0.08	[17]
Maize bran	68.80 ± 0.20	21.40 ± 0.04	47.20 ± 0.09	
Oat bran	61.20 ± 0.30	16.20 ± 0.05	47.20 ± 0.09	
Pea	10.40 ± 2.33	1.73 ± 0.26	20.30 ± 0.40	[18]
Common bean	8.55 ± 3.31	2.42 ± 0.74	19.90 ± 0.19	
Chickpea	9.88 ± 2.11	0.00 ± 0.00	13.90 ± 0.09	
Lentil	6.83 ± 2.42	1.44 ± 0.11	19.00 ± 1.27	
Lyophilized plum skin	38.98 ± 0.52	14.19 ± 0.66	24.81 ± 1.16	[19]
Pomegranate (Acide)	28.27 ± 0.90	1.16 ± 0.11	27.11 ± 0.65	[20]
Actinidia deliciosa	92.88 ± 0.75	32.85 ± 0.24	60.03 ± 0.50	[21]
Kiwifruit pomace	56.44 ± 1.02	6.90 ± 0.20	49.54 ± 0.86	[22]
Okara	65.96 ± 0.05	2.24 ± 0.10	63.72 ± 0.18	[23,24]
Rose pomace	46.30 ± 0.40	4.45 ± 0.81	41.12 ± 1.15	[25]

**Table 2:** The functional enhancement methods of Plant-sourced Dietary Fibers.

Source of PDF	Method	Enhancement Activity	Improvement of Beneficial Bioactivity
Barley β-glucan [102,103]	β-glucanase hydrolysis	Improving viscosity	Increased yeast gas production and indirectly improve gluten network structure.
Wheat bran [104]	Cold plasma and enzymatic hydrolysis	Enhancing lipid-lowering ability	The combined modified SDF showed the best performance in glucose adsorption capacity, cholesterol adsorption capacity and antioxidant capacity.
Litchi Pomace [105,106]	Ultrasonic-enzymatic co-modification	Enriching beneficial bacteria	The modified SDF significantly stimulated the growth of probiotic bacterial species.
[107]	Extrusion and cellulase modification	Exposing more surface area and functional groups	The physicochemical properties and in vitro functional activity were enhanced, while the Pb^2+^ adsorption capacity was strengthened.
Rose pomace [25]	Enzymatic hydrolysis (EH) and ultrasound-assisted enzymatic hydrolysis (UEH)	Enhancing the looseness of the surface structure, resulting in a shale-like blocky appearance	Enhanced the capacity of oil-holding, swelling, cation-exchange, and cholesterol adsorption capacities.
Okara [108]	*K. marxianu* fermentation and β-glucosidase hydrolysis	Increasing porosity and reducing bulk density	Enhanced and prolonged the adsorption capacity for both glucose and cholesterol.
Orange pomace [109]	Extrusion processing	Increasing the content of soluble dietary fiber	Increased the uronic acid content in the SDF fraction.
Defatted rice bran [110]	γ-irradiation combined with enzymatic modification	Reducing particle size and increasing specific surface area	Enhanced the anti-digestive properties and probiotic activity

## Data Availability

No new data were created or analyzed in this study.

## Abbreviations

The following abbreviations are used in this manuscript:

PDF	Plant-sourced Dietary Fiber
